# Draft genome sequence of *Nonomuraea bangladeshensis* strain B208 with high potential for specific cytochrome P450 monooxygenases

**DOI:** 10.1128/mra.01371-25

**Published:** 2026-02-18

**Authors:** Hikaru Suenaga, Kentaro Miyazaki, Toshikazu Fukushima

**Affiliations:** 1 Cellular and Molecular Biotechnology Research Institute, National Institute of Advanced Industrial Science and Technology (AIST), Tsukuba, Japan; 2 International Center for Biotechnology, The University of Osaka 13013, Suita, Osaka, Japan; 3 Environment Research Laboratory, Advanced Technology Research Laboratories, Nippon Steel Corporation, Futtsu, Chiba, Japan; Montana State University, Bozeman, Montana, USA

**Keywords:** *Nonomuraea*, cytochrome P450, flavonoid, biotransformation, activared sludge

## Abstract

*Nonomuraea bangladeshensis* B208 has been reported to convert several flavonoids into hydroxylated derivatives. Here, we present a draft genome sequence that reveals the gene sets encoding putative cytochrome P450 monooxygenases associated with the generation of oxygenated flavonoids of medical and nutritional relevance.

## ANNOUNCEMENT

Hydroxylation of C‒H bonds catalyzed by cytochrome P450 monooxygenases (CYPs) can lead to the formation of high-value compounds used as specialty chemicals and pharmaceutical intermediates, a transformation that is generally difficult to achieve using conventional organic chemistry (1). Bioprocesses proceed under ambient temperature and pressure conditions, reducing CO₂ emissions relative to high-temperature, high-pressure chemical syntheses (2). Therefore, the isolation and characterization of novel CYPs from microbial resources are crucial for the development of industrial biotransformation processes (3). Comprehensive genetic screening of promising microbial strains substantially expands the repertoire of specific CYPs, thereby enabling access to entirely new classes of compounds and enhancing the efficiency of established biotransformations (4, 5). The antioxidative properties of flavonoids have been extensively studied due to their medical and nutritional significance (6, 7). *Nonomuraea bangladeshensis* B208 was isolated from activated sludge used in coke-oven wastewater treatment as previously described (8). This strain was shown to convert several flavonoids, such as naringen and pinocembrin, into hydroxylated derivatives (data not shown). Here, we analyzed the draft genome sequence with a special focus on identifying its repertoire of CYP genes.

Strain B208 was cultivated in ISP2 medium (yeast extract 4 g/L, malt extract 10 g/L, and glucose 4 g/L) at 28°C for 3 days. The genomic DNA was isolated with a Qiagen DNeasy genomic DNA kit in combination with a Genomic-tip 20G according to the manufacturer’s protocol (Qiagen K.K., Venlo, the Netherlands) and sheared into an average fragment size of 7‒15 kb using a Megaruptor 3 system (Diagenode SA, Seraing, Belgium). Sequencing was performed on the Revio platform with the PacBio Revio Polymerase Kit (Pacific Biosciences of California, Menlo Park, CA, USA). The genome library was prepared using the SMRTbell Prep Kit 3.0 (Pacific Biosciences of California) and the Twist Universal Adapter System (Twist Bioscience, South San Francisco, CA, USA). The sequencer produced 32,022 reads with an average read length of 7,743 bp. Low-quality reads and reads <1,000 bp were trimmed using Filtlong v0.21. The sequence reads were assembled using Flye v2.9.3-b1797. The assembled genome consists of 35 contigs totaling 10,182,372 bp, with a G + C content of 71.9% and 24.4× coverage. The N50 contig size and the largest contig size were 521,825 and 1,233,325 bp, respectively. CheckM2 v.1.0.1 estimated the genome to be 100.0% complete with 0.84% contamination. All software for gene assembly and genome analysis was used with default parameters.

Taxonomic assignment was performed using the DDBJ Fast Annotation and Submission Tool v1.3.6 (9). Based on the average nucleotide identity (ANI), strain B208 was identified as *Nonomuraea bangladeshensis* (ANI 98.36%), exceeding the widely accepted cutoff of 95%–97% (10). Gene functional annotation using Prokka v1.0.1 identified 9,767 coding genes, including 15 rRNA and 78 tRNA genes. The predicted protein sequences were compared with sequences in the Kyoto Encyclopedia of Genes and Genomes (KEGG) database and the Gene Ontology (GO) database (geneontology.org/) using the BLASTP command implemented in Diamond v2.1.6 to assign KEGG IDs and GO terms. Additional annotation identified 32 CYP genes. The identified gene sequences are candidates for heterologous expression and characterization, with potential application in the biotransformation of diverse flavonoid compounds.

## Data Availability

All data have been deposited in DDBJ/ENA/GenBank under the following accession numbers: BioProject PRJDB37998, BioSample SAMD01704683, Sequence Read Archive DRR793359, and the assembled and annotated genome accessions BAAJOU010000001–BAAJOU010000035.
